# Differential attrition and engagement in randomized controlled trials of occupational mental health interventions in person and online: A systematic review and meta-analysis

**DOI:** 10.5271/sjweh.4173

**Published:** 2024-12-01

**Authors:** Carlota de Miquel, Josep Maria Haro, Christina M van der Feltz-Cornelis, Ana Ortiz-Tallo, Tom Chen, Marjo Sinokki, Päivi Naumanen, Beatriz Olaya, Rodrigo A Lima

**Affiliations:** 1 Research, Innovation and Teaching Unit, Parc Sanitari Sant Joan de Déu, Sant Boi de Llobregat, Spain.; 2 Centro de Investigación Biomédica en Red de Salud Mental (CIBERSAM), Madrid, Spain.; 3 Department of Medicine, Universitat de Barcelona, Barcelona, Catalunya, Spain.; 4 Dept. of Health Sciences, Hull York Medical School, University of York, York, Country.; 5 Institute of Health Informatics, University College London, London, United Kingdom; 6 Department of Psychiatry, School of Medicine, Universidad Autónoma de Madrid, Madrid, Spain.; 7 Kent Business School, University of Kent, Canterbury, United Kingdom; 8 Canberra Business School, University of Canberra, Canberra, Australia.; 9 The National Centre for Epidemiology and Population Health, College of Health and Medicine, The Australian National University, Canberra, Australia.; 10 Länsirannikon Työterveys Oy, Turku, Finland.; 11 Unit of Public Health, University of Turku, Finland.; 12 University of Turku, Department of Education, Turku, Finland

**Keywords:** drop-out, employee, methodology, psychology

## Abstract

**Objective:**

This study systematically reviewed and meta-analyzed the differential attrition and utilization of occupational mental health interventions, specifically examining delivery methods (internet-based versus in-person).

**Methods:**

The research, with papers spanning 2010–2024, involved filtering criteria and comprehensive searches across PubMed, Scopus, and Web of Science Core (PROSPERO registration n. CRD42022322394). Of 28 683 titles, 84 records were included in the systematic review, with 75 in meta-analyses. Risk of bias was assessed through the revised Cochrane risk of bias tool for randomized control trials and funnel plots. Differential attrition across studies was meta-analysed through a random-effects model with limited maximum-likelihood estimation for the degree of heterogeneity.

**Results:**

Findings reveal higher mean differential attrition in the intervention group, indicating a potential challenge in maintaining participant engagement. The attrition rates were not significantly influenced by the mode of intervention delivery (internet versus in-person). Compensation for participation and year of publication could potentially influence differential attrition from baseline to follow-up measurements.

**Conclusions:**

These results suggest a need for cautious consideration of attrition in occupational mental health intervention study designs and emphasize the importance of adapting statistical analyses to mitigate potential bias arising from differential attrition.

The toll that mental illnesses take on individuals, families, companies, and economies, governments and non-governmental organizations around the world has been widely recognized. Mental health issues impact millions of people worldwide and are among the top causes of years lived with disability globally (1). They not only lead to disability but also contribute to unemployment and dependence on welfare benefits (2). Indeed, in 2010, the estimated yearly costs of mental illness were $2.5 trillion globally (3) and, in 2016, more than €450 billion for the European Union (4). It is expected that this number will rise to $6 trillion globally by 2030 (5), as indirect expenses related to mental disorders, such as sick leave days, have recently increased (6). As a result, promoting and addressing mental health in the workplace has gained strategic importance, with expected advantages for employees and companies.

Health promotion interventions in the workplace have been found to be successful in preventing mental health issues (7), with reviews also showing the effectiveness of in-person and online therapies in preventing and reducing mental health issues (8–11). However, these reviews also reported high heterogeneity between studies with respect to the approaches used for these interventions, their outcomes, and their methodological quality (10, 11). One of the challenges reported for digital mental interventions is the high rates of attrition, which is often under investigated.

Studies using return-to-work interventions, for example, showed high attrition rates (uneven attrition between intervention and control groups and significant loss to follow-up) and did not routinely assess compliance with the intervention (12). This was also seen in a systematic review of digital mental health interventions in the workplace (13), where many studies reported high levels of attrition, but where 11 of 32 studies failed to adequately describe any procedures for managing attrition or missing values, and where 11 (of 32) studies failed to provide power calculations for their sample sizes. A systematic review of universal and targeted workplace interventions for depression found specifically computerized interventions to have the highest mean attrition rate compared with other delivery methods (14). Low adherence and study attrition are regular and serious issues that could compromise the reliability of the results (15), therefore there is need to examine when and why these phenomena takes place.

The patient characteristics and trial factors that influence the overall uptake of an intervention can be learned by analyzing dropout rates and when they occur (16). Differential attrition, which is viewed as a serious threat to internal validity, occurs when attrition rates vary between treatment conditions (17). In a health behavior change trial, a slightly higher amount of attrition on average was found in the intervention conditions as compared to the control groups (18). Similar results were found for mHealth randomized control trials, where attrition in active conditions was, on average, roughly twice than that of controls (19). The authors speculated that the reason for higher attrition in treatment groups in mHealth might be an increased burden associated with their intervention. Another meta-analysis on smartphone-delivered mHealth interventions evaluated the different factors that affected attrition. It concluded that trials (i) delivering an acceptance-based intervention, (ii) providing participants with a financial reward, and (iii) reminding individuals to participate in the intervention had lower attrition rates compared to trials that used an online enrolment strategy (eg, by telephone instead of in-person enrolment) (15). Indeed, no baseline participant-level trait accurately predicted attrition. As far as we are aware, no meta-analysis has previously examined differential attrition in mental health interventions in the workplace and how this might vary across internet- and in-person-based interventions.

Therefore, the aim of this study was to systematically review and meta-analyze differential attrition of occupational mental health interventions, differentiating by delivery method (ie, internet- versus in-person based). The secondary research question of this meta-analysis was to assess which factors might be related to differential attrition in such studies.

## Methods

This study was a systematic review with a meta-analysis, which followed the PRISMA statement (20). The study protocol was prospectively published in PROSPERO (CRD42022322394), and work was conducted under the EMPOWER (European Platform to Promote Well-being and Health in the Workplace) project, funded by the European Commission (21). The EMPOWER project researches the impact of an eMental health platform on preventing common mental health problems and reducing psychological distress in the workplace.

### Search methods

On 1 February 2022, we conducted a search in the electronic databases of PubMed, Scopus and Web of Science Core. The searches were performed filtered by year range (2010–2022), type of study (randomized control trial, RCT), species (human), age (adult) and language (English, Spanish, Portuguese) when possible. To structure the eligibility criteria, the PICOS (Patient/Population; Intervention; Comparison, Outcome; Study design) approach was used. The combination of words from five different areas was used, namely mental health, intervention, workplace, implementation and study design. A full list of the search words can be found in the supplementary material (sjweh.fi/article/4173), appendix A. Additionally, experts from the EMPOWER Consortium were consulted for potential additional references during the months of January and February of 2023. An additional search was conducted in April 2024 following the same criteria.

### Study population and article selection

The study population included adult employees who participated in a study focusing on preventing or reducing mental health problems in the workplace. We included those studies focusing on any kind of mental health problem except for addictions. Therefore, interventions were included if they aimed at promoting employee’s mental health in the workplace or reducing employee’s mental health symptoms. Additionally, studies were only included if they: (i) followed a RCT design, with control and intervention groups; (ii) included digital mental health interventions or traditional, non-digital mental health interventions or interventions containing both modalities that were implemented in a workplace environment for ≥6 weeks; (iii) reported response rates at baseline and/or attrition rates at post-treatment/follow-up or provided information that allowed us to calculate them; (iv) were published between 2010 and the search date and the publication language was either English, Spanish or Portuguese; (v) recruited participating employees from the workplace itself and the primary outcome was measured in employees.

**Figure 1:**
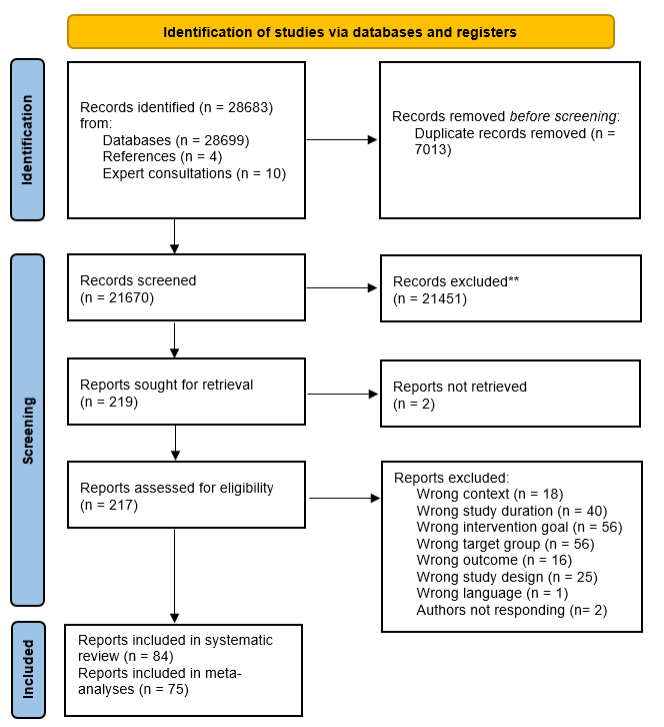
PRISMA flowchart diagram

Six reviewers collaborated on the selection of studies, hence two researchers independently reviewed 25% of the studies by title and abstract. After title and abstract screening, two researchers reviewed the manuscripts’ full-text for inclusion in the review. Researchers were blinded to each other’ decisions. A disagreement between individual judgments were resolved by a third independent party for the title and abstract screening and by a discussion between the reviewers for the full-text screening (see figure 1). Rayyan software was used throughout the study selection process. Prior to the screening, the six researchers evaluated a subset of eligible manuscripts to ensure compliance with inclusion and exclusion criteria.

### Data extraction

Two researchers independently extracted the following data for selected studies via consensus methods: title and year of publication, aims and purpose, study population, country of study implementation, sample size, study methodology/methods, name of mental health intervention, main outcomes, intervention format, assessment and intervention times, setting/context, usage data of the intervention, attrition, number of participants contacted to participate in the study and number who agreed to participate, as well as attrition and non-participation reasons.

### Quality assessment

The revised Cochrane risk of bias tool for randomized control trials (ROB 2) (22), was used to assess the quality of included trials. Two researchers (CM and RL) independently assessed each study according to screening questions with the five prescribed domains on risk of bias: arising from the randomization process; due to deviations from the intended interventions assignment to intervention); due to missing outcome data; in measurement of the outcome; and in selection of the reported result. Each screening question was assigned a low, medium, or high score, and then an overall risk of bias score was calculated based on these domains. Results are visually presented in supplementary table S1.

### Statistical analysis

For each study, we created a 2×2 table with the number of participants who were lost to post-intervention and follow-up and the number who remained in the study in the intervention condition and control condition. After aggregating the available data, we determined the percentage of participants in each study’s intervention and control conditions who were lost to follow-up after randomization using the 2×2 tables. The risk ratio, where a value >1 indicates a higher attrition rate in the intervention condition and a value <1 indicates a higher attrition rate in the control condition, was the outcome of interest for the meta-analysis. In order to produce values that are symmetric around zero and whose sample distribution is better approximated by a normal distribution, the actual analysis was conducted using the log-transformed risk ratios. We then meta-analyzed the log-transformed relative attrition rates using a random-effects model with limited maximum-likelihood (REML) estimation for the degree of heterogeneity (23). Along with the outcomes of the *Q*-test for heterogeneity and the *I*^2^ statistic, we also present the (back-transformed) estimated average relative attrition rate and accompanying 95% confidence interval (CI). The funnel plot’s asymmetry was visually inspected for potential signs of publishing bias.

The link between the log-transformed relative attrition rates and various study parameters that may be associated to the degree of differential attrition was examined using meta-regression analysis with mixed-effects models using REML estimation (23). For baseline to post-intervention differential attrition, the following potential effect moderators were explored: time to post-intervention measure, length of the intervention, type of control group, compensation for participation, type of country according to the World Bank classification (low and middle income versus high income countries), type of intervention (in-person versus online), engagement restrictions, type of workers (white or blue collar), sample size at baseline, intervention (psychosocial, physical activity, occupational or multilevel), the specificity of the intervention (general mental health versus specific mental health concern), quality of the study, and if the inclusion criteria for the sample was suffering from mental health problems (ie, scoring above a certain threshold in a mental health diagnostic tool). For baseline to follow-up attrition, we calculated the potential effect of the same moderators, only modifying the time to post-intervention measure to the time to the last follow-up measure. The various variables were estimated univariately. All analyses were conducted in R (24) using the metafor package (25).

## Results

### Study characteristics

A total of 28 683 studies were included following the literature search and reference check (figure 1). After removal of duplicates, 21 670 were identified for abstract and title screening. Subsequently, 217 were included in the full-text screening from which 84 were included in the systematic review and 75 in the meta-analysis. The main reasons for exclusion in the full-text screening were different intervention goals, target group and study duration. Nine records were not included in the meta-analyses for the following reasons: one study did not report attrition rates (26), one study contained more participants at post-intervention than at baseline (27) and seven studies reported the same data in two papers (28–34).

A summary of the study characteristics can be found in supplementary table S2. The search resulted on 46 studies based on in-person delivered interventions, 29 studies of interventions delivered online, and 6 studies delivered through other formats. The studies encompass a wide range of target outcomes, including stress, depression, anxiety, burnout, and general mental health well-being. The mean participation rate was 42.51% [standard deviation (SD) 29.46%, range 1.27–100%], with a mean of 36.18% (SD 27.92%, range 1.27–92.5%) in online delivered interventions, 49.85% (SD 29.68%, range 2.08–100%) for in-person delivered interventions, and 13.69% (SD 1.94%, range 11.52–15.27%) for interventions delivered through other methodologies (eg, blended interventions or phone interventions). Most of the studies (N=42) used wait-list control group whereas 32 maintained the participants’ usual care as a control condition. Other control conditions were active control (6) and wait-list active control (2).

### Qualitative analyses of the results

The reasons for attrition more often reported were lack of time (35–41), job situation change or work-conflict (37, 41–55) and personal health issues (45, 56, 57). In some studies, participants were excluded if they did not complete a certain percentage of the intervention [eg (40, 58–60),]. In the majority of the studies not all participants underwent the complete intervention [eg (42, 44, 61, 62),], and in many cases, some participants allocated in the intervention group did not participate in the intervention at all [eg (63–65),]. Additionally, many studies did not report usage data [eg (62, 66–68),].

### Baseline to post-intervention differential attrition analyses

The average total attrition from baseline to post-intervention measure was 17.59% (SD 17.25, range 0–69.29%), with an average attrition of 19.77% (SD 20.08%, range 0–75.02%) for the intervention group and 15.68% (SD 16.25%, range 0–65.99%) for the control group. The random effects model showed a significantly higher attrition in the intervention compared to control group with a pooled risk ratio of 1.03 (see figure 2). In the case of online interventions, the average total attrition was 27.53% (SD 21.54%, range 0–75.02%), the average attrition in the intervention group was 31.74% (SD 25.62%, range 0–75.02%) and 24.51% (SD 20.63%, range 0–65.99%) in the control group. The random effects model online interventions also showed a higher attrition in the intervention group as compared to the control group with a pooled risk ratio of 1.04. In interventions delivered in person, the average total attrition was 12.93% (SD=11.99%, range=0–41.27%), the average attrition in the intervention group was 14.45% (SD=13.53%, range=0–47.49%) and in the control group was 11.50% (SD=11.56%, range=0–36.84%). The random effects model of in-person-delivered interventions did not show a higher attrition in the intervention compared to control group. Lastly, for interventions delivered through other methodologies, the average total attrition was 13.49% (SD 15.90%, range 0–40.45%), the average attrition in the intervention group was 13.37% (SD 17.25%, range 0–43.51%) and 13.30% (SD 14.41%, range 0–35.96%) in the control group. For other types of intervention, the random effects model did not result in a significant differential attrition. However, there was not a significant difference in differential attrition between interventions delivered online, in-person or through other methodologies. A summarized description of the main results can be found in supplementary table S3.

**Figure:**
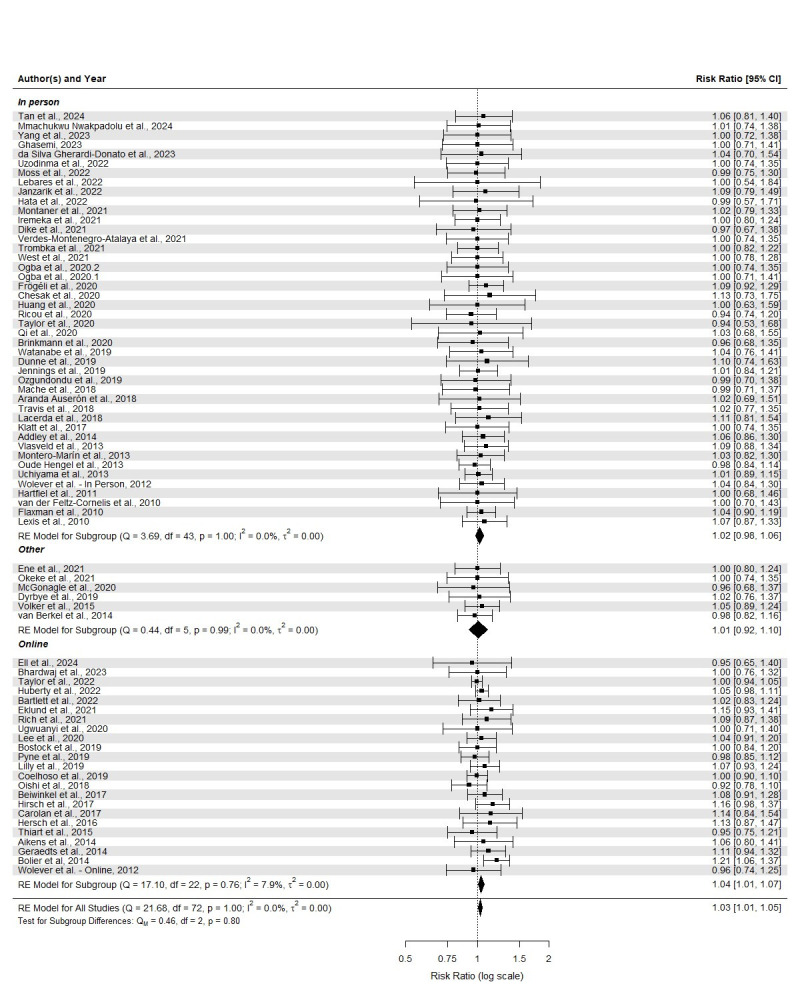
**Figure 2.** Forest plot for meta-analysis and subgroup meta-regression for baseline to post-intervention differential attrition.

**Figure 3:**
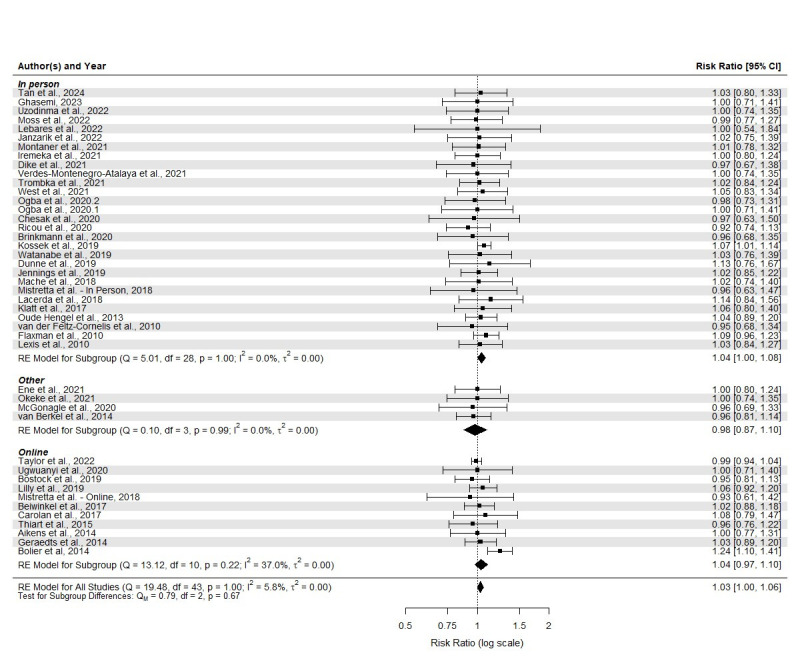
Forest plot for meta-analysis and subgroup metaregression for baseline to last follow-up differential attrition.

Meta-regression univariate analyses showed no significant effects of any moderator variable (see supplementary tables S4 and S5). Mean attrition values from baseline to post-intervention for the different categorical moderators can also be found in supplementary table S6.

### Baseline to follow-up differential attrition analyses

The average total attrition from baseline to follow up measure was 22.25% (SD 17.97%, range 0–67.78%), with an average attrition of 22.35% (SD 18.90%, range 0–69%) for the intervention group and 21.38% (SD 17.67%, range 0–66.25%) for the control group. The random effects model showed a higher attrition in the intervention compared to control group with a pooled risk ratio of 1.03. In the case of online interventions, the average total attrition was 32.12% (SD 19.43%, range 0–67.78%), the average attrition in the intervention group was 29.71% (SD 21.48%, range 0–69%) and 30.40% (SD 18.96%, range 0–66.25%) in the control group. The random effects model for online interventions did not show a higher attrition in the intervention compared to control group. In interventions delivered in person, the average total attrition was 20.21% (SD 16.60%, range 0–59.16%), the average attrition in the intervention group was 21.64% (SD 17.35%, range 0–64.41%) and 21.29% (SD 17.52%, range 0–64.41%) in the control group. The random effects model of in-person-delivered interventions showed a significantly higher attrition in the intervention compared to control group with a pooled risk ratio of 1.04. Lastly, for interventions delivered through other methodologies, the average total attrition was 14.54% (SD 17.46%, range 0–33.9%), the average attrition in the intervention group was 11.26% (SD 14.48%, range 0–27.59%) and 17.07% (SD 19.25%, range 0–37.93%) in the control group. For other types of intervention, the random effects model did not result in a significant differential attrition. However, there was not a significant difference in differential attrition between interventions delivered online, in-person and through other methodologies. A summarized description of the main results can be found in supplementary table S3.

Two of the tested moderators were found to significantly predict differential attrition from baseline to follow-up, namely compensation for participation and year of publication (see supplementary tables S4 and S5). Mean attrition values from baseline to follow-up for the different categorical moderators can also be found in supplementary table S6).

### Publication bias

**Figure 4:**
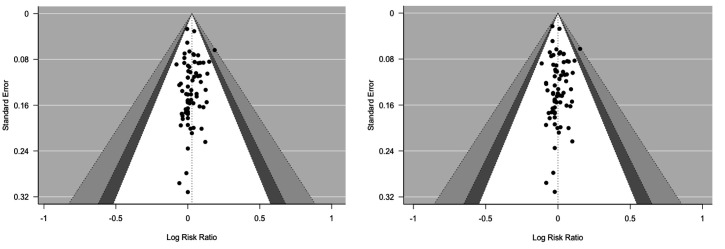
Funnel plots for publication bias. In the left side for the baseline to post-intervention attrition measurements and in the right side the baseline to follow-up attrition measurements

As can be seen in the funnel plots (figure 4), no study seems to be highly affected by publication bias.

## Discussion

In this meta-analysis, we examined differential attrition in occupational mental health interventions. Compared to the control group, participants in the intervention arm exhibited higher differential attrition from baseline to post-intervention and from baseline to the latest follow-up measurement. These results line up with previous meta-analysis on differential attrition in behavioral and clinical interventions (18, 19). The fact that participants in occupational mental health treatments are generally not blinded for the treatment allocation could be a speculative reason for the somewhat higher attrition rates in the intervention group. Participants may have consequently higher expectations for perceived treatment efficacy. If these expectations are not satisfied, participants in the intervention conditions may be less likely to complete follow-up assessments than participants in the control conditions who may have lower expectations to begin with. However, when attrition reasons were reported in studies, the reasons mentioned were rather lack of time (35–39), job situation change or work-conflict (37, 42–52) and personal health issues (45, 56, 57).

In the current meta-analysis, we did not observe a difference in the differential attrition rates between in-person and internet-based interventions. Indeed, when analyzed separately, differential attrition was only significant in internet-based interventions for the baseline to post-assessment measurement. However, differential attrition from baseline to follow-up assessment was found to only be significant for in-person interventions. Moreover, when compared, no significant difference was encountered between the differential attrition of both intervention types. Therefore, it seems that differential attrition affects occupational mental health intervention generally rather than the modality of intervention delivery.

When examining the potential moderators of differential attrition, we found that none of them were connected to the degree of differential attrition when looking at attrition from baseline to post-intervention. Nevertheless, for baseline to follow-up interventions, we could see a trend for a significant moderator effect for participants being compensated for their participation and year of publication. Variables such as time to follow-up or type of control condition were also examined in other meta-analyses and also failed to have a significant effect of the variables on differential attrition (18, 69). However, in a previous meta-analysis (15) the authors found an effect of financial compensation on differential attrition, aligning with our results. Even if the moderators evaluated in the meta-analysis already address many potential variations between trials (such as treatment length, length of evaluation period, type of intervention, compensation), there might be other additional factors that play a role in explaining differential attrition. Future research should expand the search for moderators to other potential variables that impact differential attrition. Identifying such variables could help tackle them and decrease differential attrition in studies and consequently increasing internal validity.

Another outcome of interest is the percentage of total attrition across studies. We encountered a mean attrition for baseline to post-intervention of 17.59% and of 22.25% from baseline to follow-up. When separating for in-person interventions, the attrition resulted 12.93% for baseline to post-intervention and 20.21% for baseline to follow-up. This is in line with previous literature on behavioral health interventions (18). For digital interventions, we found a mean attrition percentage of 27.53% from baseline to post-intervention and of 32.12% for baseline to follow-up. This outcome also aligns with previous findings in smartphone-delivered interventions for mental health problems (15), which show a mean percentage attrition of 24.1% for short-term follow-up and 35.5% at longer-term follow-up. These results would also be in line with a previous systematic review of universal and targeted workplace interventions for depression, which concluded that digital interventions had the highest mean attrition rate when compared with other delivery methods (14). Moreover, these values can serve as a reference for future studies.

This systematic review and meta-analysis has some limitations that must be taken into account. First, our subgroup analyses on study-related factors were based on studies that used distinct samples with varying occupations and different interventions, targeting different mental health issues. Therefore, it is not obvious whether these findings may be applied to certain target mental health problems or particular interventions and occupations. While we added moderator analyses controlling for the effect of the intervention delivered or whether the goal of the intervention was to improve mental health in general or a specific mental health condition, our analyses may not have captured the effect of the heterogeneity of the studies included. Additionally, the results of this study are limited to occupational mental health interventions published from 2010 onwards and in certain languages, which may lead to publishing and cultural biases in our results.

To conclude, our research suggests that differential attrition rates are frequent and might be problematic in RCTs for occupational mental health interventions. The findings have a number of consequences for scientists, decision-makers, and clinicians. First, it is reasonable to assume that between one-quarter and one-third of participants in future trials of smartphone therapies might drop out from the study. When creating recruiting plans and doing a priori power analysis, this amount of lost data should be taken into account to ensure that the RCT’s statistical validity is not jeopardized. Moreover, statistical analyses should be adapted accordingly. When differential attrition takes place, a usual complete cases analysis would lead to biased results, whereas using an expectation-maximization algorithm could tackle this bias if the attrition mechanism is accessible (70). Although our results were unable to shed light on the type of participants most likely to drop-out from the RCT, there may be some strategies that researchers might apply to potentially reduce attrition (eg, use personalized enrollment methods, gamification of the intervention). Moreover, as we found that compensating for the participation may influence attrition, this could potentially be a strategy to increase study adherence in occupational mental health intervention studies. For well-being and mental health interventions through mobile apps, gamification can be a useful and effective platform. It may also increase motivation and decrease attrition (71).

## Supplementary material

Supplementary material

## References

[1] Vos T, Barber RM, Bell B, Bertozzi-Villa A, Biryukov S, Bolliger I et al.; Global Burden of Disease Study 2013 Collaborators. Global, regional, and national incidence, prevalence, and years lived with disability for 301 acute and chronic diseases and injuries in 188 countries, 1990-2013: a systematic analysis for the Global Burden of Disease Study 2013. Lancet 2015 Aug;386(9995):743–800. 10.1016/S0140-6736(15)60692-4 26063472 PMC4561509

[2] Nicholson PJ. Common mental disorders and work. Br Med Bull 2018 Jun;126(1):113–21. 10.1093/bmb/ldy014 29684103

[3] Bloom DE, Cafiero E, Jané-Llopis E, Abrahams-Gessel S, Bloom LR, Fathima S et al. The Global Economic Burden of Noncommunicable Diseases. Geneva: World Economic Forum; 2011;(September).

[4] European Commission. EU Joint Action Mental Health and Wellbeing. European Framework for Action on Mental Health and Wellbeing. 2016 [cited 2024 Jun 12]. Available from: https://ec.europa.eu/research/participants/data/ref/h2020/other/guides_for_applicants/h2020-SC1-BHC-22-2019-framework-for-action_en.pdf

[5] World Economic Forum, Harvard School of Public Health. Methodological Appendix: The Global Economic Burden of Non-Communicable Diseases. World Economic Forum. 2011;(September).

[6] Tiainen A, Rehnberg C. The economic burden of psychiatric disorders in Sweden. Int J Soc Psychiatry 2010 Sep;56(5):515–26. 10.1177/0020764009106140 19734184

[7] Proper KI, van Oostrom SH. The effectiveness of workplace health promotion interventions on physical and mental health outcomes - a systematic review of reviews. Scand J Work Environ Health 2019 Nov;45(6):546–59. 10.5271/sjweh.3833 31134284

[8] Carolan S, Harris PR, Cavanagh K. Improving employee well-being and effectiveness: systematic review and meta-analysis of web-based psychological interventions delivered in the workplace. J Med Internet Res 2017 Jul;19(7):e271. 10.2196/jmir.7583 28747293 PMC5550734

[9] Stratton E, Lampit A, Choi I, Calvo RA, Harvey SB, Glozier N. Effectiveness of eHealth interventions for reducing mental health conditions in employees: A systematic review and meta-analysis. PLoS One 2017 Dec;12(12):e0189904. 10.1371/journal.pone.0189904 29267334 PMC5739441

[10] Bhui KS, Dinos S, Stansfeld SA, White PD. A synthesis of the evidence for managing stress at work: a review of the reviews reporting on anxiety, depression, and absenteeism. J Environ Public Health 2012;2012:515874. 10.1155/2012/515874 22496705 PMC3306941

[11] Corbière M, Shen J, Rouleau M, Dewa CS. A systematic review of preventive interventions regarding mental health issues in organizations. Work 2009;33(1):81–116. 10.3233/WOR-2009-0846 19597288

[12] Cullen KL, Irvin E, Collie A, Clay F, Gensby U, Jennings PA et al. Effectiveness of Workplace Interventions in Return-to-Work for Musculoskeletal, Pain-Related and Mental Health Conditions: An Update of the Evidence and Messages for Practitioners. J Occup Rehabil 2018 Mar;28(1):1–15. 10.1007/s10926-016-9690-x 28224415 PMC5820404

[13] Armaou M, Araviaki E, Dutta S, Konstantinidis S, Blake H. Effectiveness of Digital Interventions for Deficit-Oriented and Asset-Oriented Psychological Outcomes in the Workplace: A Systematic Review and Narrative Synthesis. Eur J Investig Health Psychol Educ 2022 Oct;12(10):1471–97. 10.3390/ejihpe12100102 36286087 PMC9601105

[14] Wan Mohd Yunus WM, Musiat P, Brown JS. Systematic review of universal and targeted workplace interventions for depression. Occup Environ Med 2018 Jan;75(1):66–75. 10.1136/oemed-2017-104532 29074553

[15] Linardon J, Fuller-Tyszkiewicz M. Attrition and adherence in smartphone-delivered interventions for mental health problems: A systematic and meta-analytic review. J Consult Clin Psychol 2020 Jan;88(1):1–13. 10.1037/ccp0000459 31697093

[16] Heneghan C, Perera R, Ward A A, Fitzmaurice D, Meats E, Glasziou P. Assessing differential attrition in clinical trials: self-monitoring of oral anticoagulation and type II diabetes. BMC Med Res Methodol 2007 May;7:18. 10.1186/1471-2288-7-18 17474976 PMC1876242

[17] Shadish WR, Cook TD, Campbell DT. Experimental and Quasi-Experimental Designs for Generalized Causal Inference. Prancan K, editor. Boston, USA: Houghton Mifflin Compan; 2001.

[18] Crutzen R, Viechtbauer W, Spigt M, Kotz D. Differential attrition in health behaviour change trials: a systematic review and meta-analysis. Psychol Health 2015 Jan;30(1):122–34. 10.1080/08870446.2014.953526 25109224

[19] Goldberg SB, Bolt DM, Davidson RJ. Data missing not at random in mobile health research: assessment of the problem and a case for sensitivity analyses. J Med Internet Res 2021 Jun;23(6):e26749. 10.2196/26749 34128810 PMC8277392

[20] Page MJ, McKenzie JE, Bossuyt PM, Boutron I, Hoffmann TC, Mulrow CD et al. The PRISMA 2020 statement: an updated guideline for reporting systematic reviews. BMJ 2021 Mar;372(71):n71. 10.1136/bmj.n71 33782057 PMC8005924

[21] Olaya B, Van der Feltz-Cornelis CM, Hakkaart-van Roijen L, Merecz-Kot D, Sinokki M, Naumanen P et al.; EMPOWER Consortium. Study protocol of EMPOWER: A cluster randomized trial of a multimodal eHealth intervention for promoting mental health in the workplace following a stepped wedge trial design. Digit Health 2022 Oct;8:20552076221131145. 10.1177/20552076221131145 36276189 PMC9583218

[22] Sterne JA, Savović J, Page MJ, Elbers RG, Blencowe NS, Boutron I et al. RoB 2: a revised tool for assessing risk of bias in randomised trials. BMJ 2019 Aug;366:l4898. 10.1136/bmj.l4898 31462531

[23] Raudenbush SW. Analyzing effect sizes: Random-effects models. In: Cooper H, Hedges LV, Valentine JC. The handbook of research synthesis and meta-analysis. 2^nd^ ed. New York: Russell Sage Foundation; 2009. p. 295–315.

[24] R Core Team. R: A Language and Environment for Statistical Computing. Vienna: R Foundation for Statistical Computing. 2021.

[25] Viechtbauer W. Conducting meta-analisys in R with metafor package. J Stat Softw 2010;36(3):1–48. 10.18637/jss.v036.i03

[26] Flook L, Goldberg SB, Pinger L, Bonus K, Davidson RJ. Mindfulness for teachers: A pilot study to assess effects on stress, burnout and teaching efficacy. Mind Brain Educ 2013 Sep;7(3): 10.1111/mbe.12026 24324528 PMC3855679

[27] Dyrbye LN, West CP, Richards ML, Ross HJ, Satele D, Shanafelt TD. A randomized, controlled study of an online intervention to promote job satisfaction and well-being among physicians. Burn Res 2016;3(3):69–75. 10.1016/j.burn.2016.06.002

[28] Qi M, Moyle W, Jones C, Weeks B. Effects of Tai Chi Combined With Theraband Training on Physical Fitness, Psychological Well-being, and Pain in Older Sedentary Office Workers. Top Geriatr Rehabil 2019 Oct;35(4):255–65. 10.1097/TGR.0000000000000244

[29] Sampson M, Melnyk BM, Hoying J. Intervention Effects of the MINDBODYSTRONG Cognitive Behavioral Skills Building Program on Newly Licensed Registered Nurses’ Mental Health, Healthy Lifestyle Behaviors, and Job Satisfaction. J Nurs Adm 2019 Oct;49(10):487–95. 10.1097/NNA.0000000000000792 31517756

[30] Volker D, Zijlstra-Vlasveld MC, Brouwers EP, van der Feltz-Cornelis CM. Process Evaluation of a Blended Web-Based Intervention on Return to Work for Sick-Listed Employees with Common Mental Health Problems in the Occupational Health Setting. J Occup Rehabil 2017 Jun;27(2):186–94. 10.1007/s10926-016-9643-4 27150734 PMC5405094

[31] Thiart H, Ebert DD, Lehr D, Nobis S, Buntrock C, Berking M et al. Internet-based cognitive behavioral therapy for insomnia: A health economic evaluation. Sleep 2016 Oct;39(10):1769–78. 10.5665/sleep.6152 27450686 PMC5020359

[32] Obiweluozo PE, Dike IC, Ogba FN, Elom CO, Orabueze FO, Okoye-Ugwu S et al. Stress in teachers of children with neuro-developmental disorders: effect of blended rational emotive behavioral therapy. Sci Prog 2021 Oct;104(4):368504211050278. 10.1177/00368504211050278 34783626 PMC10402289

[33] Akanaeme IN, Ekwealor FN, Ifeluni CN, Onyishi CN, Obikwelu CL, Ohia NC et al. Managing job stress among teachers of children with autism spectrum disorders: A randomized controlled trial of cognitive behavioral therapy with yoga. Medicine (Baltimore) 2021 Nov;100(46):e27312. 10.1097/MD.0000000000027312 34797272 PMC8601364

[34] Avallone Mantelli R, Forster J, Edelblute A, Sinn H, Torres K, Adams T et al. Creative Arts Therapy for Healthcare Professionals Is Associated With Long-Term Improvements in Psychological Distress. J Occup Environ Med 2023 Dec;65(12):1032–5. 10.1097/JOM.0000000000002963 37705403

[35] Geraedts AS, Kleiboer AM, Wiezer NM, van Mechelen W, Cuijpers P. Short-term effects of a web-based guided self-help intervention for employees with depressive symptoms: randomized controlled trial. J Med Internet Res 2014 May;16(5):e121. 10.2196/jmir.3185 24800966 PMC4026573

[36] Lee S, Rozybakieva Z, Asimov M, Bagiyarova F, Tazhiyeva A, Ussebayeva N et al. Coping strategy as a way to prevent emotional burnout in primary care doctors: A randomized controlled trial. Arch Balk Med Union. 2020;55(3):398–409. 10.31688/ABMU.2020.55.3.04

[37] Taylor J, McLean L, Richards B, Glozier N. Personalised yoga for burnout and traumatic stress in junior doctors. Postgrad Med J 2020 Jun;96(1136):349–57. 10.1136/postgradmedj-2019-137413 32300055

[38] Trombka M, Demarzo M, Campos D, Antonio SB, Cicuto K, Walcher AL et al. Mindfulness Training Improves Quality of Life and Reduces Depression and Anxiety Symptoms Among Police Officers: Results From the POLICE Study-A Multicenter Randomized Controlled Trial. Front Psychiatry 2021 Feb;12:624876. 10.3389/fpsyt.2021.624876 33716824 PMC7952984

[39] Thiart H, Lehr D, Ebert DD, Berking M, Riper H. Log in and breathe out: internet-based recovery training for sleepless employees with work-related strain - results of a randomized controlled trial. Scand J Work Environ Health 2015 Mar;41(2):164–74. 10.5271/sjweh.3478 25590336

[40] Gherardi-Donato EC, Gimenez LB, Fernandes MN, Lacchini R, Camargo Júnior EB, Díaz-Serrano KV et al. Mindfulness Practice Reduces Hair Cortisol, Anxiety and Perceived Stress in University Workers: Randomized Clinical Trial. Healthcare (Basel) 2023 Oct;11(21):2875. 10.3390/healthcare11212875 37958019 PMC10648523

[41] Bhardwaj P, Pathania M, Bahurupi Y, Kanchibhotla D, Harsora P, Rathaur VK. Efficacy of mHealth aided 12-week meditation and breath intervention on change in burnout and professional quality of life among health care providers of a tertiary care hospital in north India: a randomized waitlist-controlled trial. Front Public Health 2023 Nov;11:1258330. 10.3389/fpubh.2023.1258330 38026380 PMC10646346

[42] Lexis MA, Jansen NW, Huibers MJ, van Amelsvoort LG, Berkouwer A, Tjin A Ton G et al. Prevention of long-term sickness absence and major depression in high-risk employees: a randomised controlled trial. Occup Environ Med 2011 Jun;68(6):400–7. 10.1136/oem.2010.057877 20924024

[43] Uchiyama A, Odagiri Y, Ohya Y, Takamiya T, Inoue S, Shimomitsu T. Effect on mental health of a participatory intervention to improve psychosocial work environment: a cluster randomized controlled trial among nurses. J Occup Health 2013;55(3):173–83. 10.1539/joh.12-0228-OA 23585499

[44] Oude Hengel KM, Blatter BM, van der Molen HF, Bongers PM, van der Beek AJ. The effectiveness of a construction worksite prevention program on work ability, health, and sick leave: results from a cluster randomized controlled trial. Scand J Work Environ Health 2013 Sep;39(5):456–67. 10.5271/sjweh.3361 23567980

[45] Klatt M, Norre C, Reader B, Yodice L, White S. Mindfulness in Motion: a Mindfulness-Based Intervention to Reduce Stress and Enhance Quality of Sleep in Scandinavian Employees. Mindfulness (N Y) 2017;8(2):481–8. 10.1007/s12671-016-0621-x

[46] Dunne PJ, Lynch J, Prihodova L, O’Leary C, Ghoreyshi A, Basdeo SA et al. Burnout in the emergency department: randomized controlled trial of an attention-based training program. J Integr Med 2019 May;17(3):173–80. 10.1016/j.joim.2019.03.009 30956141

[47] Pyne JM, Constans JI, Nanney JT, Wiederhold MD, Gibson DP, Kimbrell T et al. Heart Rate Variability and Cognitive Bias Feedback Interventions to Prevent Post-deployment PTSD: Results from a Randomized Controlled Trial. Mil Med 2019 Jan;184(1-2):e124–32. 10.1093/milmed/usy171 30020511 PMC6751385

[48] Ricou B, Gigon F, Durand-Steiner E, Liesenberg M, Chemin-Renais C, Merlani P et al. Initiative for Burnout of ICU Caregivers: Feasibility and Preliminary Results of a Psychological Support. J Intensive Care Med 2020 Jun;35(6):562–9. 10.1177/0885066618768223 29642743

[49] Chesak SS, Bhagra A, Cutshall S, Ingram A, Benoit R, Medina-Inojosa JR et al. Authentic Connections Groups: A Pilot Test of an Intervention Aimed at Enhancing Resilience Among Nurse Leader Mothers. Worldviews Evid Based Nurs 2020 Feb;17(1):39–48. 10.1111/wvn.12420 32017436

[50] Lebares CC, Hershberger AO, Guvva EV, Desai A, Mitchell J, Shen W et al. Feasibility of Formal Mindfulness-Based Stress-Resilience Training Among Surgery Interns: A Randomized Clinical Trial. JAMA Surg 2018 Oct;153(10):e182734. 10.1001/jamasurg.2018.2734 30167655 PMC6233792

[51] Lilly M, Calhoun R, Painter I, Beaton R, Stangenes S, Revere D et al. Destress 9-1-1-an online mindfulness-based intervention in reducing stress among emergency medical dispatchers: a randomised controlled trial. Occup Environ Med 2019 Oct;76(10):705–11. 10.1136/oemed-2018-105598 31138676

[52] Qi M, Moyle W, Jones C, Weeks B. Feasibility of a tai chi with thera-band training program: A pilot study. Int J Environ Res Public Health 2020 Nov;17(22):8462. 10.3390/ijerph17228462 33207580 PMC7696740

[53] Vlasveld MC, van der Feltz-Cornelis CM, Adèr HJ, Anema JR, Hoedeman R, van Mechelen W et al. Collaborative care for sick-listed workers with major depressive disorder: a randomised controlled trial from the Netherlands Depression Initiative aimed at return to work and depressive symptoms. Occup Environ Med 2013 Apr;70(4):223–30. 10.1136/oemed-2012-100793 23112266

[54] Ell J, Brückner HA, Johann AF, Steinmetz L, Güth LJ, Feige B et al. Digital cognitive behavioural therapy for insomnia reduces insomnia in nurses suffering from shift work disorder: A randomised-controlled pilot trial. J Sleep Res 2024 Mar;e14193:e14193. 10.1111/jsr.14193 38485134 PMC11596998

[55] Janzarik G, Wollschläger D, Wessa M, Lieb K. A Group Intervention to Promote Resilience in Nursing Professionals: A Randomised Controlled Trial. Int J Environ Res Public Health 2022 Jan;19(2):649. 10.3390/ijerph19020649 35055470 PMC8775927

[56] Addley K, Boyd S, Kerr R, McQuillan P, Houdmont J, McCrory M. The impact of two workplace-based health risk appraisal interventions on employee lifestyle parameters, mental health and work ability: results of a randomized controlled trial. Health Educ Res 2014 Apr;29(2):247–58. 10.1093/her/cyt113 24399261

[57] Dike IC, Onyishi CN, Adimora DE, Ugodulunwa CA, Adama GN, Ugwu GC et al. Yoga complemented cognitive behavioral therapy on job burnout among teachers of children with autism spectrum disorders. Medicine (Baltimore) 2021 Jun;100(22):e25801. 10.1097/MD.0000000000025801 34087823 PMC8183729

[58] Montero-Marín J, Asún S, Estrada-Marcén N, Romero R, Asún R. [Effectiveness of a stretching program on anxiety levels of workers in a logistic platform: a randomized controlled study]. Aten Primaria 2013;45(7):376–83. 23764394 10.1016/j.aprim.2013.03.002 PMC6985483

[59] Aikens KA, Astin J, Pelletier KR, Levanovich K, Baase CM, Park YY et al. Mindfulness goes to work: impact of an online workplace intervention. J Occup Environ Med 2014 Jul;56(7):721–31. 10.1097/JOM.0000000000000209 24988100

[60] Coelhoso CC, Tobo PR, Lacerda SS, Lima AH, Barrichello CR, Amaro E Jr et al. A new mental health mobile app for well-being and stress reduction in working women: randomized controlled trial. J Med Internet Res 2019 Nov;21(11):e14269. 10.2196/14269 31697244 PMC6873146

[61] van Berkel J, Boot CR, Proper KI, Bongers PM, van der Beek AJ. Effectiveness of a worksite mindfulness-related multi-component health promotion intervention on work engagement and mental health: results of a randomized controlled trial. PLoS One 2014 Jan;9(1):e84118. 10.1371/journal.pone.0084118 24489648 PMC3904825

[62] Volker D, Zijlstra-Vlasveld MC, Anema JR, Beekman AT, Brouwers EP, Emons WH et al. Effectiveness of a blended web-based intervention on return to work for sick-listed employees with common mental disorders: results of a cluster randomized controlled trial. J Med Internet Res 2015 May;17(5):e116. 10.2196/jmir.4097 25972279 PMC4468600

[63] Bolier L, Ketelaar SM, Nieuwenhuijsen K, Smeets O, Gärtner FR, Sluiter JK. Workplace mental health promotion online to enhance well-being of nurses and allied health professionals: A cluster-randomized controlled trial. Internet Interv 2014;1(4):196–204. 10.1016/j.invent.2014.10.002

[64] Geraedts AS, Kleiboer AM, Twisk J, Wiezer NM, van Mechelen W, Cuijpers P. Long-term results of a web-based guided self-help intervention for employees with depressive symptoms: randomized controlled trial. J Med Internet Res 2014 Jul;16(7):e168. 10.2196/jmir.3539 25008127 PMC4115257

[65] Hersch RK, Cook RF, Deitz DK, Kaplan S, Hughes D, Friesen MA et al. Reducing nurses’ stress: A randomized controlled trial of a web-based stress management program for nurses. Appl Nurs Res 2016 Nov;32:18–25. 10.1016/j.apnr.2016.04.003 27969025 PMC5159423

[66] Wolever RQ, Bobinet KJ, McCabe K, Mackenzie ER, Fekete E, Kusnick CA et al. Effective and viable mind-body stress reduction in the workplace: a randomized controlled trial. J Occup Health Psychol 2012 Apr;17(2):246–58. 10.1037/a0027278 22352291

[67] Mistretta EG, Davis MC, Temkit M, Lorenz C, Darby B, Stonnington CM. Resilience Training for Work-Related Stress Among Health Care Workers: Results of a Randomized Clinical Trial Comparing In-Person and Smartphone-Delivered Interventions. J Occup Environ Med 2018 Jun;60(6):559–68. 10.1097/JOM.0000000000001285 29370014

[68] Kossek EE, Thompson RJ, Lawson KM, Bodner T, Perrigino MB, Hammer LB et al. Caring for the elderly at work and home: can a randomized organizational intervention improve psychological health? J Occup Health Psychol 2019 Feb;24(1):36–54. 10.1037/ocp0000104 29215909 PMC5991990

[69] W B, A S, P J, Ga J, Tj W; W B. Systematic review and meta analysis of differential attrition between active and control arms in randomized controlled trials of lifestyle interventions in chronic disease. BMC Med Res Methodol 2021 Jun;21(1):122. 10.1186/s12874-021-01313-x 34126934 PMC8204467

[70] Graham JW, Donaldson SI. Evaluating interventions with differential attrition: the importance of nonresponse mechanisms and use of follow-up data. J Appl Psychol 1993 Feb;78(1):119–28. 10.1037/0021-9010.78.1.119 8449850

[71] Litvin S, Saunders R, Maier MA, Lüttke S. Gamification as an approach to improve resilience and reduce attrition in mobile mental health interventions: A randomized controlled trial. PLoS One 2020 Sep;15(9):e0237220. 10.1371/journal.pone.0237220 32877425 PMC7467300

[72] Flaxman PE, Bond FW. Worksite stress management training: moderated effects and clinical significance. J Occup Health Psychol 2010 Oct;15(4):347–58. 10.1037/a0020522 21058850

[73] van der Feltz-Cornelis CM, Hoedeman R, de Jong FJ, Meeuwissen JA, Drewes HW, van der Laan NC et al. Faster return to work after psychiatric consultation for sicklisted employees with common mental disorders compared to care as usual. A randomized clinical trial. Neuropsychiatr Dis Treat 2010 Sep;6:375–85. 10.2147/NDT.S11832 20856601 PMC2938286

[74] Hartfiel N, Havenhand J, Khalsa SB, Clarke G, Krayer A. The effectiveness of yoga for the improvement of well-being and resilience to stress in the workplace. Scand J Work Environ Health 2011 Jan;37(1):70–6. 10.5271/sjweh.2916 20369218

[75] Carolan S, Harris PR, Greenwood K, Cavanagh K. Increasing engagement with an occupational digital stress management program through the use of an online facilitated discussion group: results of a pilot randomised controlled trial. Internet Interv 2017 Aug;10:1–11. 10.1016/j.invent.2017.08.001 30135747 PMC6084816

[76] Hirsch A, Luellen J, Holder JM, Steinberg G, Dubiel T, Blazejowskyj A et al. Managing depressive symptoms in the workplace using a web-based self-care tool: A pilot randomized controlled trial. JMIR Res Protoc 2017 Apr;6(4):e51. 10.2196/resprot.7203 28377368 PMC5395692

[77] Beiwinkel T, Eißing T, Telle NT, Siegmund-Schultze E, Rössler W. Effectiveness of a web-based intervention in reducing depression and sickness absence: randomized controlled trial. J Med Internet Res 2017 Jun;19(6):e213. 10.2196/jmir.6546 28619701 PMC5491897

[78] Oishi S, Takizawa T, Kamata N, Miyaji S, Tanaka K, Miyaoka H. Web-based training program using cognitive behavioral therapy to enhance cognitive flexibility and alleviate psychological distress among schoolteachers: pilot randomized controlled trial. JMIR Res Protoc 2018 Jan;7(1):e32. 10.2196/resprot.8541 29374006 PMC5807627

[79] Lacerda SS, Little SW, Kozasa EH. A stress reduction program adapted for the work environment: A randomized controlled trial with a follow-up. Front Psychol 2018 May;9:668. 10.3389/fpsyg.2018.00668 29867646 PMC5954607

[80] Travis F, Valosek L, Konrad A 4th, Link J, Salerno J, Scheller R et al. Effect of meditation on psychological distress and brain functioning: A randomized controlled study. Brain Cogn 2018 Aug;125:100–5. 10.1016/j.bandc.2018.03.011 29936408

[81] Aranda Auserón G, Elcuaz Viscarret MR, Fuertes Goñi C, Güeto Rubio V, Pascual Pascual P. Sainz de Murieta García de Galdeano E [Evaluation of the effectiveness of a Mindfulness and Self-Compassion program to reduce stress and prevent burnout in Primary Care health professionals]. Aten Primaria 2018;50(3):141–50. 28629886 10.1016/j.aprim.2017.03.009 PMC6836985

[82] Mache S, Bernburg M, Baresi L, Groneberg D. Mental health promotion for junior physicians working in emergency medicine: evaluation of a pilot study. Eur J Emerg Med 2018 Jun;25(3):191–8. 10.1097/MEJ.0000000000000434 27879536

[83] Dyrbye LN, Shanafelt TD, Gill PR, Satele DV, West CP. Effect of a Professional Coaching Intervention on the Well-being and Distress of Physicians: A Pilot Randomized Clinical Trial. JAMA Intern Med 2019 Oct;179(10):1406–14. 10.1001/jamainternmed.2019.2425 31380892 PMC6686971

[84] Ozgundondu B, Gok Metin Z. Effects of progressive muscle relaxation combined with music on stress, fatigue, and coping styles among intensive care nurses. Intensive Crit Care Nurs 2019 Oct;54:54–63. 10.1016/j.iccn.2019.07.007 31371164

[85] Jennings PA, Doyle S, Oh Y, Rasheed D, Frank JL, Brown JL. Long-term impacts of the CARE program on teachers’ self-reported social and emotional competence and well-being. J Sch Psychol 2019 Oct;76:186–202. 10.1016/j.jsp.2019.07.009 31759466

[86] Bostock S, Crosswell AD, Prather AA, Steptoe A. Mindfulness on-the-go: effects of a mindfulness meditation app on work stress and well-being. J Occup Health Psychol 2019 Feb;24(1):127–38. 10.1037/ocp0000118 29723001 PMC6215525

[87] Watanabe N, Horikoshi M, Shinmei I, Oe Y, Narisawa T, Kumachi M et al. Brief mindfulness-based stress management program for a better mental state in working populations - Happy Nurse Project: A randomized controlled trial^✰✰^. J Affect Disord 2019 May;251:186–94. 10.1016/j.jad.2019.03.067 30927579

[88] McGonagle AK, Schwab L, Yahanda N, Duskey H, Gertz N, Prior L et al. Coaching for primary care physician well-being: A randomized trial and follow-up analysis. J Occup Health Psychol 2020 Oct;25(5):297–314. 10.1037/ocp0000180 32297776

[89] Brinkmann AE, Press SA, Helmert E, Hautzinger M, Khazan I, Vagedes J. Comparing Effectiveness of HRV-Biofeedback and Mindfulness for Workplace Stress Reduction: A Randomized Controlled Trial. Appl Psychophysiol Biofeedback 2020 Dec;45(4):307–22. 10.1007/s10484-020-09477-w 32556709 PMC7644531

[90] Sampson M, Melnyk BM, Hoying J. The MINDBODYSTRONG Intervention for New Nurse Residents: 6-Month Effects on Mental Health Outcomes, Healthy Lifestyle Behaviors, and Job Satisfaction. Worldviews Evid Based Nurs 2020 Feb;17(1):16–23. 10.1111/wvn.12411 31721425

[91] Huang L, Harsh J, Cui H, Wu J, Thai J, Zhang X et al. A Randomized Controlled Trial of Balint Groups to Prevent Burnout Among Residents in China. Front Psychiatry 2020 Feb;10:957. 10.3389/fpsyt.2019.00957 32116808 PMC7026367

[92] Frögéli E, Rudman A, Ljótsson B, Gustavsson P. Preventing Stress-Related Ill Health Among New Registered Nurses by Supporting Engagement in Proactive Behaviors-A Randomized Controlled Trial. Worldviews Evid Based Nurs 2020 Jun;17(3):202–12. 10.1111/wvn.12442 32592439

[93] Ogba FN, Onyishi CN, Ede MO, Ugwuanyi C, Nwokeoma BN, Victor-Aigbodion V et al. Effectiveness of SPACE Model of Cognitive Behavioral Coaching in Management of Occupational Stress in a Sample of School Administrators in South-East Nigeria. J Ration-Emot Cogn-B. 2020;38(3):345–68. 10.1007/s10942-019-00334-2

[94] Ogba FN, Onyishi CN, Victor-Aigbodion V, Abada IM, Eze UN, Obiweluozo PE et al. Managing job stress in teachers of children with autism: A rational emotive occupational health coaching control trial. Medicine (Baltimore) 2020 Sep;99(36):e21651. 10.1097/MD.0000000000021651 32898998 PMC7478671

[95] Ugwuanyi CS, Okeke CI, Agboeze MU, Igwe NJ, Eya NM, Ejimonye JC et al. Impacts of cognitive behavior therapy on occupational stress among science and social science education facilitators in open and distance learning centers and its implications for community development: A randomized trial group. Medicine (Baltimore) 2020 Oct;99(41):e22677. 10.1097/MD.0000000000022677 33031335 PMC7544423

[96] West CP, Dyrbye LN, Satele DV, Shanafelt TD. Colleagues Meeting to Promote and Sustain Satisfaction (COMPASS) Groups for Physician Well-Being: A Randomized Clinical Trial. Mayo Clin Proc 2021 Oct;96(10):2606–14. 10.1016/j.mayocp.2021.02.028 34366134

[97] Rich RM, Ogden J, Morison L. A randomized controlled trial of an app-delivered mindfulness program among university employees: effects on stress and work-related outcomes. Int J Workplace Health Manag 2021;14(2):201–16. 10.1108/IJWHM-04-2020-0046

[98] Verdes-Montenegro-Atalaya JC, Pérula-de Torres LÁ, Lietor-Villajos N, Bartolomé-Moreno C, Moreno-Martos H, Rodríguez LA et al.; On Behalf Of The Minduudd Collaborative Study Group. Effectiveness of a mindfulness and self-compassion standard training program versus an abbreviated training program on stress in tutors and resident intern specialists of family and community medicine and nursing in Spain. Int J Environ Res Public Health 2021 Sep;18(19):10230. 10.3390/ijerph181910230 34639532 PMC8507764

[99] Eklund C, Söderlund A, Elfström ML. Evaluation of a Web-Based Stress Management Program for Persons Experiencing Work-Related Stress in Sweden (My Stress Control): Randomized Controlled Trial. JMIR Ment Health 2021 Dec;8(12):e17314. 10.2196/17314 34889772 PMC8704112

[100] Ene CU, Ugwuanyi CS, Ejimonye JC, Ani MI, Eneogu ND, Ikeh FE et al. Effects of rational emotive occupational health coaching on work stress among academic staff of science and social science education in Nigerian universities: A randomised trial evaluation. Medicine (Baltimore) 2021 Aug;100(34):e26963. 10.1097/MD.0000000000026963 34449461 PMC8389868

[101] Iremeka FU, Okeke SA, Agu PU, Isilebo NC, Aneke M, Ezepue EI et al. Intervention for stress management among skilled construction workers. Medicine (Baltimore) 2021 Jul;100(28):e26621. 10.1097/MD.0000000000026621 34260549 PMC8284712

[102] Okeke FC, Onyishi CN, Nwankwor PP, Ekwueme SC. A blended rational emotive occupational health coaching for job-stress among teachers of children with special education needs. Internet Interv 2021 Nov;26:100482. 10.1016/j.invent.2021.100482 34824983 PMC8604685

[103] Montaner X, Tárrega S, Pulgarin M, Moix J. Effectiveness of Acceptance and Commitment Therapy (ACT) in Professional Dementia Caregivers Burnout. Clin Gerontol 2022;45(4):915–26. 10.1080/07317115.2021.1920530 33955318

[104] Bartlett L, Martin AJ, Kilpatrick M, Otahal P, Sanderson K, Neil AL. Effects of a Mindfulness App on Employee Stress in an Australian Public Sector Workforce: Randomized Controlled Trial. JMIR Mhealth Uhealth 2022 Feb;10(2):e30272. 10.2196/30272 35142630 PMC8874803

[105] Hata SR, Berkowitz LR, James K, Simpkin AL. An Interprofessional Group Intervention to Promote Faculty Well-Being: A Randomized Clinical Trial. J Contin Educ Health Prof 2022 Jan;42(1):e75–82. 10.1097/CEH.0000000000000404 34799518

[106] Huberty JL, Espel-Huynh HM, Neher TL, Puzia ME. Testing the Pragmatic Effectiveness of a Consumer-Based Mindfulness Mobile App in the Workplace: Randomized Controlled Trial. JMIR Mhealth Uhealth 2022 Sep;10(9):e38903. 10.2196/38903 36169991 PMC9557765

[107] Moss M, Edelblute A, Sinn H, Torres K, Forster J, Adams T et al. The Effect of Creative Arts Therapy on Psychological Distress in Health Care Professionals. Am J Med 2022 Oct;135(10):1255–1262.e5. 10.1016/j.amjmed.2022.04.016 35576997

[108] Taylor H, Cavanagh K, Field AP, Strauss C. Health Care Workers’ Need for Headspace: Findings From a Multisite Definitive Randomized Controlled Trial of an Unguided Digital Mindfulness-Based Self-help App to Reduce Healthcare Worker Stress. JMIR Mhealth Uhealth 2022 Aug;10(8):e31744. 10.2196/31744 36006668 PMC9459942

[109] Uzodinma UE, N Onyishi C, Ngwoke AN, Ugwu JI, Okorie CO, A Amujiri B et al. Effectiveness of rational emotive occupational health coaching in reducing burnout symptoms among teachers of children with autism. Sci Prog 2022;105(2):368504221100907. 10.1177/00368504221100907 35619571 PMC10450314

[110] Ghasemi F. A randomized controlled trial of an adapted group cognitive-behavioral therapy for burned-out teachers. Psychother Res 2023 Apr;33(4):494–507. 10.1080/10503307.2022.2131476 36404282

[111] Yang F, Fei Y, Guo L, Bai X, Li X. Job crafting intervention for job burnout and work engagement among young construction project management practitioners in China. Eng Constr Archit Ma.2023. doi: 10.1108/ECAM-10-2022-0935. [Epub ahead of print.] 10.1108/ECAM-10-2022-0935

[112] Nwakpadolu GM, Ede MO, Okoro JO, Nwadi CL, Akudo FU, Anigbogu GN et al. Effect of psychological intervention in cushioning work-induced stress among secondary school home economics teachers: implications for policy and administration. Medicine (Baltimore) 2024 Mar;103(9):e37174. 10.1097/MD.0000000000037174 38428875 PMC10906576

[113] Tan L, Deady M, Mead O, Foright RM, Brenneman EM, Bryant RA et al. Yoga resilience training to prevent the development of posttraumatic stress disorder in active-duty first responders: A cluster randomized controlled trial. Psychol Trauma 2024 Mar;•••: ; [Epub ahead of print]. 10.1037/tra0001667 38451716

