# Supplementary material for: Differential attrition and engagement in randomized controlled trials of occupational mental health interventions in person and online: A systematic review and meta-analysis
Source: Scand J Work Environ Health. 2024 Nov 28;50(8):588–601. doi: 10.5271/sjweh.4173 (PMC11616721; doi:10.5271/sjweh.4173)
Supplement: Supplementary material [file SJWEH-50-588-S001.pdf]

Differential attrition and engagement in randomized controlled trials of occupational mental health interventions in person and online: A systematic review and meta-analysis[1]

by Carlota de Miquel, MSc, Josep Maria Haro, PhD, Christina M van der Feltz-Cornelis, PhD, Ana Ortiz-Tallo, MSc, Tom Chen, PhD, Marjo Sinokki, PhD, Päivi Naumanen, PhD, Beatriz Olaya, PhD, Rodrigo A Lima, PhD

1. Supplementary material
2. Correspondence to: Beatriz Olaya, Carrer Doctor Antoni Pujadas 42, 08830 Sant Boi de Llobregat, Barcelona, Spain. [E-Mail: beatriz.olaya@sjd.es]

Appendix A – Search Terms

((mental disorder*) OR (mental health) OR (mental illness*) OR (well-being) OR anxiety OR burnout OR depressed OR depression OR depressive OR mindfulness OR psycholog* OR stress OR wellbeing OR absenteeism OR presenteeism OR (mental problem*) OR (work function*) OR productivity OR resilience OR (work impairment))

AND

(computer-based* OR (mobile application*) OR (remote consultation*) OR app OR apps OR computer-aided OR digital OR ehealth OR e-health OR emental OR e-mental OR internet-based OR online OR on-line OR smartphone-based OR web-based OR (whatsapp intervention) OR intervention OR (health care) OR therap* OR promot* OR prevent* OR treatment OR treat* OR psychotherapy OR (stress management) OR (cognitive behavioral therapy) OR CBT OR (in-person based) OR (in-person treat*))

AND

(business* OR companies OR company OR employe* OR employment* OR enterprise OR job OR jobs OR occupation OR occupational OR work OR worker OR workers OR working OR work-life OR workload* OR workplace* OR worksite* OR worksite-based OR workspace*)

AND

(implement OR implementation OR adoption OR utilisation OR uptake OR (program evaluation) OR implemented OR implementing OR up-take OR (process evaluation) OR sustain OR sustainability OR engage OR engagement)

AND

(RCT OR (Randomized Controlled Trial))

Table S1. Risk of bias and quality assessment

	Risk of bias domains					
	D1	D2	D3	D4	D5	Overall
Lexis et al. 2010 [42]	+	-	+	+	-	-
Flaxman & Bond 2010 [72]	+	+	+	+	-	-
van der Ferltz-Cornelis et al. 2010 [73]	+	-	+	+	+	-
Hartfiel et al. 2011 [74]	+	X	-	+	-	X
Wolever et al. 2012 [66]	+	+	+	+	-	-
Uchiyama et al. 2013 [43]	+	X	+	+	+	X
Hengel et al. 2013 [44]	-	+	+	+	+	-
Montero-marín et al. 2013 [58]	+	+	+	+	+	+
Flook et al. 2013 [26]	+	-	+	+	X	-
Vlasveld et al. 2013 [53]	+	-	+	+	+	-
Bolier et al. 2014 [63]	-	-	-	+	+	X
Geraedts et al. 2014 [64]	+	-	-	+	+	-
Van Berkel et al. 2014 [61]	+	-	+	+	+	-
Addley et al. 2014 [56]	+	X	-	+	-	X
Aikens et al. 2014 [59]	+	-	+	+	-	-
Dyrbye et al. 2016 [27]	+	-	+	+	-	-
Hersch et al. 2016 [65]	+	-	-	+	-	X
Thiart et al. 2015 [39] & 2016 [31]	+	-	+	+	+	-
Volker et al. 2015 [62] & 2017 [30]	+	-	+	+	+	-
Carolan et al. 2017 [75]	+	-	+	+	+	-
Hirsch et al. 2017 [76]	+	-	-	+	-	X
Klatt et al. 2017 [45]	+	-	+	+	-	-
Beiwinkel et al. 2017 [77]	+	-	+	+	+	-
Oishi et al. 2018 [78]	+	-	-	+	-	X
Lacerda et al. 2018 [79]	+	X	-	+	+	X
Travis et al. 2018 [80]	+	X	+	+	-	X
Aranda Auserón et al. 2018 [81]	+	X	+	+	-	X
Mistretta et al. 2018 [67]	-	-	+	+	-	X
Mache et al. 2018 [82]	+	X	+	+	-	X
Coelhoso et al. 2019 [60]	+	-	-	+	+	-
Lilly et al. 2019 [51]	+	-	-	+	+	-
Dyrbye et al. 2019 [83]	+	-	+	+	+	-
Ozgundondu et al. 2019 [84]	+	-	+	+	-	-
Jennings et al. 2019 [85]	-	X	-	+	-	X
Dunne et al. 2019 [46]	+	X	-	+	-	X
Pyne et al. 2019 [47]	-	-	-	-	-	X
Bostock et al. 2019 [86]	+	X	+	+	-	X

Bostock et al. 2019 [86]	+	X	+	+	-	X
Watanabe et al. 2019 [87]	+	-	+	+	+	-
Kossek et al. 2019 [68]	+	-	+	+	-	-
McGonagle et al. 2020 [88]	+	X	+	+	-	X
Brinkmann et al. 2020 [89]	+	-	+	+	+	-
Qi et al. 2019 [28] & 2020 [52]	+	-	+	+	+	-
Taylor et al. 2020 [37]	+	X	-	+	-	X
Sampson et al. 2019 [29] & 2020 [90]	+	X	+	+	-	X
Ricou et al. 2020 [48]	+	-	-	+	X	X
Huang et al. 2020 [91]	+	X	-	+	-	X
Lee et al. 2020 [36]	+	X	+	+	-	X
Chesak et al. 2020 [49]	+	X	+	+	-	X
Frögéli et al. 2020 [92]	+	-	+	+	-	-
Ogba et al. 2020a [93]	+	-	+	+	+	-
Ogba et al. 2020b [94]	+	-	+	+	-	-
Ugwuanyi et al. 2020 [95]	+	-	+	+	-	-
West et al. 2021 [96]	+	-	+	+	-	-
Rich et al. 2021 [97]	+	-	+	+	-	-
Trombka et al. 2021 [38]	+	-	+	+	+	-
Verdes-Montenegro-Atalaya et al. 2021 [98]	+	-	-	+	+	-
Eklund et al. 2021 [99]	-	-	-	+	-	X
Ene et al. 2021 [100]	+	-	+	+	-	-
Iremeka et al. 2021 [101]	+	-	+	+	-	-
Okeke et al. 2021 [102] & Obiweluozo et al. 2022 [32]	+	-	+	+	-	-
Dike et al. 2021 [57] & Akanaeme et al. 2021 [33]	+	-	+	+	-	-
Montaner et al. 2021 [103]	+	+	+	+	-	-
Bartlett et al. 2022 [104]	+	+	-	+	+	-
Hata et al. 2022 [105]	-	-	-	+	-	X
Huberty et al. 2022[106]	-	-	-	+	-	X
Janzarik et al. 2022 [55]	+	-	-	+	-	X
Lebares et al. 2022 [50]	+	-	-	-	+	X
Moss et al. 2022 [107] & Mantelli et al. 2023 [34]	X	+	+	+	+	X
Taylor et al. 2022 [108]	+	+	-	+	-	-
Uzodinma et al. 2022 [109]	+	+	+	+	X	X
Bhardwaj et al. 2023 [41]	-	+	-	+	+	-
Gherardi-Donato et al. 2023 [40]	+	-	-	+	+	-
Ghasemi, 2023 [110]	-	+	+	+	-	-
Yang et al. 2023 [111]	+	-	-	+	-	X
Ell et al. 2024 [54]	+	-	+	+	+	-
Nwakpadolu et al. 2024 [112]	-	-	+	+	X	X
Tan et al. 2024 [113]	X	-	-	+	+	X

Domains:
D1: Bias arising from the randomization process.
D2: Bias due to deviations from intended intervention.
D3: Bias due to missing outcome data.
D4: Bias in measurement of the outcome.
D5: Bias in selection of the reported result.

Judgement
X High
- Some concerns
+ Low

Table S2. Study characteristics

Paper	Country	Type of intervention	Treatment length(weeks)	Main outcome(s)	Control group	Sample size
Lexis et al. 2010 (42)	The Netherlands	In person	26	Sickness absence and depressive complains	Usual care	139
Flaxman & Bond 2010 (72)	United Kingdom	In person	15	General health	Wait-list	311
Van der Feltz-Cornelis et al. 2010 (73)	The Netherlands	In person	Individual	Return to work, quality of life, and depressive, anxiety, or somatoform symptoms	Usual care	60
Hartfiel et al. 2011 (74)	United Kingdom	In person	6	Well-being and resilience to stress	Wait-list	48
Wolever et al. 2012 (66)	USA	Both	12	Stress	Usual care	239
Uchiyama et al. 2013 (43)	Japan	In person	26	Mental health status and psychosocial work environment	Wait-list	401
Hengel et al. 2013 (44)	The Netherlands	In person	26	Work ability and health	Usual care	293
Montero-marín et al. 2013 (58)	Spain	In person	13	Anxiety	Usual care	134
Flook et al. 2013 (26)	USA	In person	8	Psychological distress, mindfulness, self-compassion, and burnout	Wait-list	18
Vlasveld et al. 2013 (53)	The Netherlands	In person	Individual	Depressive symptoms	Usual care	126
Bolier et al. 2014 (63)	The Netherlands	Online	4	Positive mental health	Wait-list	366
Geraedts et al. 2014 (64)	The Netherlands	Online	6 - 7	Depressive and anxiety symptoms, burnout, work performance, and healthcare utilization	Usual care	231
Van Berkel et al. 2014 (61)	The Netherlands	Other	26	Work engagement, general mental health, need for recovery, and mindfulness	Usual care	257
Addley et al. 2014 (56)	United Kingdom	Person	52	Lifestyle, mental health, and work ability	Usual care	180
Aikens et al. 2014 (59)	USA	Online	7	Mindfulness, stress, resilience, vigor, and lifestyle	Wait-list	89
Dyrbye et al. 2016 (27)	USA	Online	10	Burnout, depressive symptoms, quality of life, work engagement, meaning in work, and satisfaction at work	Usual care	276
Hersch et al. 2016 (65)	USA	Online	13	Stress	Wait-list	104
Thiart et al. 2015 (39) & 2016 (31)	Germany	Online	6	Insomnia, costs, presenteeism, and absenteeism	Usual care	128
Volker et al. 2015 (62) & 2017 (30)	The Netherlands	Other	Individual	Return to work, depressive and anxiety symptoms, and somatic symptoms	Usual care	220
Carolan et al. 2017 (75)	United Kingdom	Online	8	Engagement, depression and anxiety symptoms, stress, and well-being	Wait-list	84
Hirsch et al. 2017 (76)	USA	Online	26	Depression	Active control	153
Klatt et al. 2017 (45)	Denmark	In person	8	Stress, sleep quality, and work engagement	Wait-list	81
Beiwinkel et al. 2017 (77)	Germany	Online	12	Depression	Wait-list	180
Oishi et al. 2018 (78)	Japan	Online	13	Cognitive flexibility and subjective distress	Wait-list	240
Lacerda et al. 2018 (79)	Brazil	In person	8	Psychiatric symptoms, stress, depression, anxiety, processing speed, and mindfulness	Wait-list	77
Travis et al. 2018 (80)	USA	In person	17.4	Brain integration and mood	Wait-list	96

Aranda Auserón et al. 2018 (81)	Spain	In person	8	Mindfulness, stress, self-compassion, and burnout	Usual care	48
Mistretta et al. 2018 (67)	USA	Both	6	Depression, stress, and well-being	Active control	60
Mache et al. 2018 (82)	Germany	In person	12	Stress, burnout, emotion regulation, job satisfaction, and work engagement	Wait-list	70
Coelhoso et al. 2019 (60)	Brazil	Online	8	Stress and well-being	Wait-list	490
Lilly et al. 2019 (51)	USA and Canada	Online	7	Stress	Wait-list	323
Dyrbye et al. 2019 (83)	USA	Other	21.7	Burnout, quality of life, job satisfaction, engagement, and meaning	Wait-list	86
Ozgundondu et al. 2019 (84)	Turkey	In person	8	Stress	Active control	63
Jennings et al. 2019 (85)	USA	In person	17.4	Psychological distress, anxiety, positive and negative affect, and burnout	Wait-list	224
Dunne et al. 2019 (46)	Ireland	In person	7	Burnout	Wait-list	47
Pyne et al. 2019 (47)	USA	Online	52	Post-Traumatic Stress Disorder (PTSD)	Usual care	342
Bostock et al. 2019 (86)	United Kingdom	Online	8	Well-being, psychological distress, job strain, workplace social support, mindfulness, and blood pressure	Wait-list	238
Watanabe et al. 2019 (87)	Japan	In person	13	Depression and anxiety	Usual care	80
Kossek et al. 2019 (68)	USA	In person	17.4	Psychological distress and stress	Usual care	1524
McGonagle et al. 2020 (88)	USA	Other	13	Burnout, work stress, turnover intentions, engagement, psychological capital, compassion, job self-efficacy, and job satisfaction	Wait-list	58
Brinkmann et al. 2020 (89)	Germany	In person	6	Chronic conditions and stress	Wait-list	62
Qi et al., 2019 (28) & 2020 (52)	Australia	In person	12	Stress, depression, anxiety, pain, and physical fitness	Active control	40
Taylor et al., 2020 (37)	Australia	In person	8	Burnout, stress, and suicidality	Wait-list active	21
Sampson et al., 2019 (29) & 2020 (90)	USA	In person	8	Stress, anxiety, depression, job satisfaction, and healthy lifestyles behaviors	Usual care	88
Ricou et al. 2020 (48)	Switzerland	In person	39	Burnout	Usual care	111
Huang et al. 2020 (91)	China	In person	26	Burnout	Wait-list	36
Lee et al. 2020 (36)	Kazakhstan	Online	52	Burnout	Usual care	243
Chesak et al. 2020 (49)	USA	In person	12	Depression, anxiety, stress, burnout, and self-compassion	Usual care	36
Frögéli et al. 2020 (92)	Sweden	In person	6	Stress	Active control	239
Ogba et al. 2020a (93)	Nigeria	In person	12	Stress	Wait-list	65
Ogba et al. 2020b (94)	Nigeria	In person	12	Stress	Wait-list	87
Ugwuanyi et al. 2020 (95)	Nigeria	Online	12	Stress	Usual care	68
West et al. 2021 (96)	USA	In person	26	Satisfaction and meaning at work, quality of life, burnout, and depression	Usual care	123
Rich et al. 2021 (97)	United Kingdom	Online	8.7	Mindfulness, stress, work-life balance, job engagement, organizational citizenship behavior, curiosity, and intention to quit	Wait-list	125
Trombka et al. 2021 (38)	Brazil	In person	8	Quality of life	Wait-list	170

Verdes-Montenegro-Atalaya et al. 2021 (98)	Spain	In person	4 - 8	Stress	Usual care	165
Eklund et al. 2021 (99)	Sweden	Online	17.4	Stress	Wait-list	92
Ene et al. 2021 (100)	Nigeria	Online	12	Stress	Usual care	63
Iremeka et al. 2021 (101)	Nigeria	In person	8	Stress and work-related irrational beliefs	Usual care	160
Okeke et al. 2021 (102) & Obiweluozo et al. 2022 (32)	Nigeria	Other	12	Stress	Wait-list	87
Dike et al. 2021 (57) & Akanaeme et al. 2021 (33)	Nigeria	In person	12	Stress and Burnout	Wait-list	58
Montaner et al. 2021 (103)	Spain	In person	6	Psychological inflexibility and burnout	Wait-list	105
Bartlett et al. 2022 (104)	Australia	Online	13	Stress, depression, psychological distress, mindfulness, quality of life, psychosocial risk factors, and productivity	Wait-list	211
Hata et al. 2022 (105)	USA	In person	13	Reaction to uncertainty, emotional exhaustion, and work engagement	Active control	25
Huberty et al. 2022 (106)	USA	Online	8	Depression, anxiety, insomnia, resilience, productivity, and medical care visits	Wait-list	1029
Janzarik et al. 2022 (55)	Germany	In person	8	General health and stress	Wait-list	72
Lebares et al. 2022 (50)	USA	In person	8	Stress, mindfulness, executive function, and feasibility	Active control	21
Moss et al. 2022 (107) and Mantelli et al. 2023 (34)	USA	In person	12	Psychological distress, anxiety, depression, burnout, PTSD, and positive and negative affect	Usual care	146
Taylor et al. 2022 (108)	United Kingdom	Online	6	Depression, anxiety, stress, mental-well-being, burnout, mindfulness, and self-compassion	Active control	2182
Uzodinma et al. 2022 (109)	Nigeria	In person	12	Burnout	Wait-list	86
Bhardwaj et al. 2023 (41)	India	Online	12	Burnout and quality of life,	Wait-list	98
Gherardi-Donato et al. 2023 (40)	Brazil	In person	8	Stress, anxiety, and mindfulness	Wait-list	42
Ghasemi 2023 (110)	Iran	In person	8	Burnout	Wait-list	66
Yang et al. 2023 (111)	China	In person	10	Burnout, work engagement, and job crafting behaviours	Usual care	72
Ell et al. 2024 (54)	Germany	Online	8		Wait-list	46
Nwakpadolu et al. 2024 (112)	Nigeria	In person	8	Stress and irrational beliefs	Wait-list	81
Tan et al. 2024 (113)	USA	In person	13	PTSD	Wait-list	80

Table S3. Summary of main results

	Mean attrition M(SD)	Intervention group attrition M(SD)	Control Group attrition M(SD)	Pooled Risk Ratio RR (95%-CI)
Pre- to post-intervention results				
General model	17.59% (17.25%)	19.77% (20.08%)	15.68% (16.25%)	**1.03 (1.01, 1.05)**
In person interventions	12.93% (11.99%)	14.45% (13.53%)	11.50% (11.56%)	1.02 (0.98, 1.06)
Online interventions	27.53% (21.54%)	31.74% (25.62%)	24.51% (20.63%)	**1.04 (1.01, 1.05)**
Other Interventions	13.49% (15.90%)	13.37% (17.25%)	13.30% (14.41%)	1.01 (0.92, 1.10)
Pre-intervention to follow-up results				
General model	22.25% (17.97%)	22.35% (18.90%)	21.38% (17.67%)	**1.03 (1.00, 1.06)**
In person interventions	20.21% (16.60%)	21.64% (17.35%)	21.29% (17.52%)	**1.04 (1.00, 1.08)**
Online interventions	32.12% (19.43%)	29.71% (21.48%)	30.40% (18.96%)	1.04 (0.97, 1.10)
Other Interventions	14.54% (17.46%)	11.26% (14.48%)	17.07% (19.25%)	0.98 (0.87, 1.10)

Note. CI = Confidence Interval, M = Mean, SD = Standard Deviation. Significant results marked in bold.

Table S4. Univariable associations for baseline to post-intervention measurement differential attrition

Variable	***Coefficient***	***95% CI***	***P***
Time to post-intervention measure	-0.000	-0.002 to 0.002	0.72
Length of the intervention	-0.000	-0.002 to 0.002	0.90
Usual care control group	0.006	-0.060 to 0.071	0.87
Waitlist control group	0.022	-0.033 to 0.078	0.43
Active waitlist control group	-0.074	-0.656 to 0.509	0.80
Compensation	-0.035	-0.083 to 0.014	0.16
LMI countries	-0.029	-0.096 to 0.036	0.38
Year of publication	-0.003	-0.009 to 0.003	0.26
White collars	0.029	-0.050 to 0.109	0.47
White and blue collars	0.082	-0.019 to 0.182	0.11
Engagement restrictions	-0.002	-0.074 to 0.071	0.97
Percentage of women	-0.000	-0.001 to 0.001	0.79
In person intervention	-0.012	-0.059 to 0.036	0.64
Other types of intervention delivery	-0.027	-0.121 to 0.067	0.58
Unsupervised online intervention	-0.010	-0.092 to 0.072	0.81
Previous mental health complains	0.019	-0.038 to 0.076	0.51
Specific mental health target	0.001	-0.043 to 0.046	0.95
Good quality research	-0.029	-0.074 to 0.016	0.20
Psychosocial intervention	0.026	-0.048 to 0.100	0.48
Physical activity intervention	0.013	-0.126 to 0.152	0.85
Occupational intervention	-0.007	-0.205 to 0.192	0.95
N at baseline in total	0.000	-0.000 to 0.000	0.25

Table S5. Univariable associations for baseline to follow-up measurement differential attrition

Variable	*Coefficient*	*95% CI*	*P*
Time to follow-up measure	0.001	-0.001 to 0.002	0.51
Length of the intervention	0.001	-0.003 to 0.005	0.56
Usual care control	0.042	-0.026 to 0.110	0.21
Waitlist control	0.060	-0.008 to 0.128	0.08
**Compensation**	**-0.058**	**-0.111 to -0.005**	**0.03**
LMI countries	-0.021	-0.111 to 0.069	0.65
**Year of publication**	**-0.007**	**-0.014 to -0.000**	**0.049**
White collars	0.008	-0.098 to 0.114	0.89
White and blue collars	-0.018	-0.148 to 0.112	0.79
Engagement restrictions	0.013	-0.160 to 0.185	0.87
Percentage of women	0.001	-0.001 to 0.002	0.56
In person intervention	0.006	-0.055 to 0.067	0.84
Other types of intervention delivery	-0.050	-0.176 to 0.076	0.43
Unsupervised online intervention	0.031	-0.101 to 0.163	0.64
Previous mental health complains	-0.032	-0.102 to 0.038	0.37
Specific mental health target	-0.025	-0.089 to 0.039	0.44
Good quality research	-0.027	-0.098 to 0.044	0.46
Psychosocial intervention	0.014	-0.101 to 0.129	0.81
Physical activity intervention	0.025	-0.254 to 0.204	0.86
Occupational intervention	0.058	-0.073 to 0.189	0.39
N at baseline in total	0.000	-0.000 to 0.000	0.78

Note. Significant effects in bold.

Table S6. Attrition percentage by intervention subgroup categories

Subgroup	*Mean attrition percentage (SD) at post-intervention*	*Mean attrition percentage (SD) at follow-up*
Location of the intervention		
High income countries	19.53% (16.91%)	26.97% (16.40%)
LMI countries	10.13% (17.03%)	7.24% (14.57%)
Engagement restrictions		
Yes	26.62% (21.57%)	34.08% (7.62%)
No	16.33% (16.36%)	21.42% (18.23%)
Mental health focus of the intervention		
Specific	17.50% (18.11%)	19.59% (19.96%)
General	17.75% (15.99%)	25.70% (14.76%)
Quality of the study		
Good quality	15.95% (14.98%)	21.33% (19.12%)
Low quality	19.82% (19.96%)	23.81% (16.24%)
Participants with previous mental health problems		
Yes	17.97% (18.66%)	20.70% (19.51%)
No	17.41% (16.71%)	36.40% (11.50%)
Intervention focus		
Psychosocial intervention	18.16% (18.65%)	22.53% (18.48%)
Physical activity intervention	18.16% (10.21%)	30.56% (27.50%)
Occupational intervention	5.49% (2.62%)	25.96% (18.31%)
Multilevel intervention	17.80% (14.35%)	15.40% (13.60%)
Compensation for participating		
Yes	9.95% (9.51%)	14.96% (15.69%)
No	20.28% (18.58%)	24.82% (18.22%)
Type of control group		
Usual care	17.14% (16.19%)	21.81% (17.35%)
Waitlist	17.20% (17.80%)	22.65% (19.29%)
Waitlist active	14.29% (-)	-
Active	21.33% (19.78%)	21.66% (16.58%)
Type of employee		
White collar	15.33% (16.42%)	19.93% (17.13%)
Blue collar	22.05 (12.57%)	26.53% (20.50%)
White and blue collar	29.24% (21.30%)	33.29% (19.98%)
Guidance (online interventions only)		
Supervised	21.74% (19.55%)	28.55% (24.82%)
Unsupervised	32.85% (22.21%)	36.40% (11.50%)
